# Patient-Reported Outcome Measures (PROM) as A Preoperative Assessment Tool

**Published:** 2017-11-02

**Authors:** Sunghye Kim, Pamela W. Duncan, Leanne Groban, Hannah Segal, Rica Moonyeen Abbott, Jeff D. Williamson

**Affiliations:** 1 Department of Internal Medicine, Winston-Salem, NC, USA; 2 Sticht Center on Aging, Winston-Salem, NC, USA; 3 Department of Neurology, Winston-Salem, NC, USA; 4 Department of Anesthesiology, Wake Forest School of Medicine, Winston-Salem, NC, USA; 5 Fisher Center for Hereditary Cancer AND Clinical Genomics Research, Georgetown University, Washington, D.C., USA

## Abstract

**Aim of review:**

Patient-reported outcomes (PRO) on functional, social, and behavioral factors might be important preoperative predictors of postoperative outcomes. We conducted a literature review to explore associations of preoperative depression, socioeconomic status, social support, functional status/frailty, cognitive status, self-management skills, health literacy, and nutritional status with surgical outcomes.

**Methods:**

Two electronic data bases, including PubMed and Google Scholar, were searched linking either depression, socioeconomic status, social support, functional status/frailty, cognitive status, self-management skills, health literacy, or nutritional status with surgery, postoperative complications, or perioperative period within the past 2 decades.

**Recent findings:**

Preoperative depression has been linked to postoperative delirium, complications, persistent pain, longer lengths of stay, and mortality. Socioeconomic status associates with overall and cancer-free survival. Low socioeconomic status has also been connected to medication non- compliance. Social support can predict overall and cancer- free survival, as well as physical, social and emotional quality of life. Poor functional status and frailty have been related to postoperative complications, longer lengths of stay, post-discharge institutionalization, and higher costs. Preoperative cognitive impairment also associates with self-medication management errors, postoperative cognitive impairment, delirium, complications and mortality. In addition, a greater tendency for reduced adherence to preoperative medication instructions has been linked to health illiteracy. Preoperative malnutrition is prevalent and associates with postoperative morbidity.

**Conclusion:**

Efficient and effective assessments of social and behavioral determinants of health, functional status, health literacy, patient’s perception of health, and preferences for self-management may improve postoperative management and surgical outcomes, particularly among vulnerable patients undergoing elective surgery who might have subtle physical, social, or psychological deficits or challenges, otherwise missed upon routine evaluation. Patient Reported Outcome Measures (PROMs) can be used to effectively and efficiently collect these factors in the preoperative period, thereby identifying areas that can be intervened preemptively. (Partially Funded by the National Institute on Aging and the Wake Forest University Claude D. Pepper Older Americans Independence Center.)

Value-based medical care should be patient-centered - defined by the Institute of Medicine as respectful of and responsive to individual patient preferences, needs and values – and assure that patients’ values guide all clinical decisions (1). Recently, the term “person- centered care”, that focuses on the whole person instead of the patient’ s medical condition, is increasingly being utilized (2). The Center for Medicare and Medicaid has set new directions for management and reimbursement of surgical patients (3). Successful implementation of bundled care and cost control for episodic payments will require more comprehensive preoperative assessment of needs for post-discharge care that incorporates factors other than traditional medical factors and fosters improved selection of the most effective post-acute plan of care. Readmission after the seventh day post- surgical discharge associates with community- and household- level factors (4) while loss of independence after surgery associates with postoperative readmission and mortality (5). Efficient and effective assessment of the social and behavioral determinants of health, comprehensive assessment of functional status, health literacy, patient’ s perception of health, and preferences for self- management may improve the success of postoperative management of vulnerable patients undergoing surgery. The Institute of Medicine recommends that social and behavioral factors be incorporated into electronic health records as a pathway to improving care quality and safety (EHRs) (6).

Although multiple questionnaires can be used to capture these data, a more effective and consistent approach would be a patient- reported outcome measures (PROMs) tool that is integrated within the EHR. PROMs are defined as any report of the status of a patient’ s health condition that comes directly from the patient, without interpretation by a clinician or anyone else (7). PROMs have been used to guide patient-centered care, clinical decision making, and health policy rulings, and are also an important tool for learning healthcare systems. PROMs improve communication between patient and providers as well as patient satisfaction (8, 9), and they are well accepted by patients and clinicians (10). PROMs may be used to improve perioperative care, by detecting preoperative deficits of patients as well as tracking postoperative trajectories. With the focus on patient- or person- centered- care and the Center for Medicare and Medicaid’ s new direction for management and reimbursement of surgical patients (3), it is imperative to include a comprehensive needs assessment for post-discharge care within the preoperative period. Incorporating PROMs into preoperative practice may be an effective and efficient way to capture functional, social and behavioral factors and also foster improved selection of the most effective post- acute plan of care. We will review the association of functional, social and behavioral factors with various surgical outcomes. The challenge and opportunities of implementing PROMs in clinical practice will be also discussed.

## Functional, Social, and Behavioral Determinants of Health and Surgical Outcomes

Functional status and social and behavioral factors affect health outcomes. A Best Practices Guideline from the American College of Surgeons National Surgical Quality Improvement Program and the American Geriatrics Society (The ACS/AGS guideline) recommends assessing these factors, including cognitive function, functional status, history of falls, frailty, nutritional status and family and social support systems during the preoperative evaluation of geriatric patients (11). Also, the Institute of Medicine (IOM) recommends that a panel of 12 social and behavioral domains be incorporated into EHRs (6). However, the assessment of social and behavioral determinants of health (SBDH) is rarely done in the preoperative setting. Specific examples of commonly encountered SBDH that affect postoperative outcomes are discussed.

### Depression

The interaction between preoperative depression and unfavorable postoperative outcomes is well recognized. Depression prior to surgery has been linked to an increased risk of postoperative, complications, including delirium and persistent pain, longer length of hospital stay and mortality (12–14). In a study of 56,064 patients who were undergoing coronary artery bypass graft surgery, pre- surgical depression associated with combined outcomes of death or re-hospitalization for myocardial infarction, heart failure, or stroke (15). In another study of 346 patients undergoing elective abdominal surgery, baseline depression was related to moderate- to- intense acute postoperative pain (16). Additionally, in a study of 872 patients who received curative- intent surgery for colorectal cancer, low preoperative self-efficacy and high depression scores predicted worse recovery trajectories in quality of life, health status and well-being (17). The positive association between preoperative depressive symptoms and increased risk for postoperative delirium has been reported in both cardiac (18–20) and noncardiac (21, 22) surgical patients, and particularly among the elderly (12). Smith et al (23) recently extended this finding by examining how different dimensions of depression, as defined by negative affect, cognitive distress, and behavioral inactivity. Of the 3% of patients with postoperative delirium, those scoring highest in the behavioral/social inactivity category of the GDS, as opposed to the negative affect and cognitive distress sub- categories of the GDS, were more likely to experience postoperative delirium. Indeed, postoperative delirium is associated with longer hospital stays, poor functional outcomes, higher healthcare costs, and even higher rates of cognitive decline (24, 25).

Table 1 summarizes the association between depression and various postoperative outcomes. Depression screens may be quickly obtained using PROMs and the data could afford the opportunity to suggest appropriate interventions designed to limit depressive symptoms before surgery that might reduce the occurrence of delirium and lingering pain early after surgery, thereby reducing hospital stay and other adverse clinical outcomes.

### Socioeconomic Status

In a study of 2,269 patients undergoing surgery for colorectal cancer, the 5-year overall survival rates and cancer-specific survival rates after surgery were significantly higher in affluent patients (55% vs 47% and 68% vs 63% for overall and cancer- specific survival, respectively) (26). Another study reported that postoperative complications after mastectomy were higher among women with lower socioeconomic status (27). In a study of 7,425 individuals with acute myocardial infarction, 31% stopped taking at least one medication by 6 months after discharge. Financial hardship was one of the reasons for this noncompliance (28). A study reported that patients without a discounted card for prescription medicines were more likely to discontinue statin medication (HR 1.63) and fail to adhere to therapy (OR 1.6) (29). In a systematic review, copayments and higher medication costs, low educational status, and ethnic minority status were associated with poor adherence (30). Measurement of socioeconomic status can contribute to predictions of postoperative outcomes and provide opportunities to offer appropriate assistance to patients with financial or other needs.

### Social Support

Social support system can determine procedure type (31) and also affect the outcome: In a study of 23,126 patients with a carcinoid tumor, marital status was related to overall and cancer free survival (32). Family and professional support was positively associated with physical, social and emotional quality of life as well as negatively associated with stress perception among colorectal cancer patients (33). The ACS/AGS guideline (11) recommends to determine the patients’ family and social support system and refer the patient to a social worker if there is concern of insufficient support in the preoperative period.

### Functional Status and Frailty

A growing body of research shows that declines in functional status are a major driver of readmissions, morbidity, and mortality (34) (35). Similar observations have been made in surgical patients: In a prospective cohort study of 372 patients who were undergoing major abdominal surgery, preoperative physical deconditioning and depression were risk factors for delayed functional recovery (36). Among 62 patients undergoing cardiac surgery, preoperative impairment in ADLs was associated with poorer functional recovery (37).

Frailty is defined as a state of increased vulnerability resulting from aging- associated declines in reserve and function across multiple physiologic systems (38). Frailty is associated with falls, worsening morbidity, inability to perform activities of daily living (ADL), hospitalization, and death in a general population (39). In a study of 594 patients who were undergoing elective surgery, frailty associated with postoperative complications, length of stay, and institutionalization (40). Another study of 197 patients who were undergoing elective noncardiac surgery, mobility, assessed using the Mobility Assessment Tool-short form (MAT-sf), predicted postoperative complications, length of stay, and institutionalization (41). Robinson et al (42) reported that advanced frailty was associated with increased surgical hospital costs, increased cost from time to discharge to 6 months postoperatively, and increased total 6- month healthcare costs. Despite this evidence and the recommendation by the ACS/AGS guideline (11), practical models have been slow to incorporate measures of functional status and frailty into preoperative assessments. The required time commitment to measure functional status of preoperative patients is part of the reason for this delay. Assessment of functional status and frailty using PROMs can identify deficits in patients’ functional status and provide a framework for proactive initiation of physical or occupational therapy before and after surgery.

### Preoperative Cognitive Impairment

Mild cognitive impairment is estimated to be present in 3– 19% of adults over 65 years old, but often not identified until late (43). Patients with cognitive impairment are more likely to make self- medication management errors (44), and are at higher risks for postoperative cognitive impairment, delirium, complications and mortality (45) (46) (47). In a study of 594 patients undergoing elective cardiac surgery, preoperative lower mini- mental status exam (MMSE) score associated with occurrence of delirium. The regression model showed that working memory (Odds ratio (OR) 0.74 [0.61– 0.89] for each unit increase in MMSE) and delayed recall (OR 0.67 [0.5– 0.89]) domains predicted delirium (48). In another study of 181 consecutive patients who were undergoing cardiac surgery, better preoperative cognitive status reduced the risk of being discharged to a facility (RR=0.95 [0.89– 0.98]) (49). In a recent cohort study of 114 older (≥70 years old patients), lower preoperative MMSE ((OR 0.68 [0.54– 0.84] per one unit MMSE) and the occurrence of postoperative delirium (OR 9.94[2.15– 26.65]) associated with incident dementia within 5 years after cardiac surgery (50). For patients who are identified as having preoperative cognitive limitations, additional care can be offered, such as assistance with postoperative medication management through home health agencies, or discharge planning to an assisted living facility.

### Self-Management Skills

The need for self-management skills is intensified in the postoperative period. In addition to managing pre- existing comorbidities, patients must manage postoperative pain and other conditions (e.g., drains, wound). Medication management is one of the most challenging tasks for many patients. Medications are often prescribed without systematic assessment of the factors that influence a patient’ s ability to manage medications. These factors include the patient’ s health literacy, executive function to self-manage multiple medications, and access to assistance if needed for medication management.

### Health Literacy

In a report by the US Department of Education, only 22% of US adults had basic health literacy skills, defined as skills necessary to perform simple and everyday literary activities, and 14% have even lower levels of literacy skills (51). Health literacy can predict mortality, mental health and physical health in the elderly (52). In a study of 332 patients undergoing ambulatory surgeries, there was a tendency for patients with health illiteracy not to adhere to preoperative medication instructions (OR 1.9, 95% CI 0.8– 4.8) (53). Another study of 34 patients recovering from open heart surgery reported that patients’ reading skills were positively correlated with comprehension of postoperative instructions (54). Patient reported data can alert the providers to offer the patients and their families the resources required, to place needed referrals to optimize medication adherence, and to offer patient and family education and engagement for improved outcomes.

### Nutritional Status

In a study of 460 patients who underwent major elective surgery, the prevalence of malnutrition was 58– 67% and morbidity rates were significantly higher in malnourished patients, with odd ratios of 2.3–3.5 (55). In a study of 1976 patients, the incidence of incision infection was significantly higher in patients with malnutrition that was defined as having one of the four criteria: weight loss >10 % within 6 months, ; body mass index (BMI) <18.5 kg/m2 ; Subjective Global Assessment Grade C; and serum albumin <3 g/dl after radical gastric surgery for gastric cancer 21.4 % vs 15.5 %, p=0.005). In addition, the 3 year overall survival (OS 59.1 vs 75%, p<0.001) and disease free survival (DFS; 54.8% vs 72.5%, p<0.001) were significantly lower in malnourished patients (56). In a study of 148,238 Medicare enrollees (age 65 to 84) who underwent one to two level posterior lumbar fusion for degenerative pathology, malnutrition defined as having diagnosis of poor nutritional status (kwashiorkor, nutritional marasmus, or other protein – calorie malnutrition) within the 3-month period before the index procedure had increased odds of 90-day major medical complications (OR 4.24) and 1- year mortality (OR 6.16). Malnutrition was also a significant predictor of increased infection (OR 2.27) and wound dehiscence (OR 2.52) (57). From 131 elderly patients who were undergoing cardiac surgery, Ogawa et al reported that poor nutritional status, as measured by the Geriatric Nutritional Risk Index (GNRI) can predict retarded postoperative rehabilitation (58). Another study of 200 patients undergoing elective GI surgery, using nutrition risk index, nutrition risk score and bioelectrical impedance analysis, the nutritional risk score was a significant prognostic factors for the development of complications (OR 4.2 (P=0.024)) (59). Various interventions, including nutritional supplementation, pharmacologic treatment for poor appetite, or financial assistance can be offered to patients who are identified as malnourished or at- risk for malnutrition using PROMs.

## The Challenges and Opportunities for Implementation of PROMS in Clinical Practice

Despite proven and/or potential role of PROMs to assess the social and functional determinants of health in various clinical settings, the acceptance of PROMS has been slow. In a survey of 1,065 cancer care professionals, lack of specificity, lack of understanding among patients on using the instrument, time commitment filling the questionnaire were identified as areas of improvements (60). Among 1997 patients who were undergoing total joint arthroplasty, the completion of PROM survey was lower in Black or Hispanic and older age, but introduction of electronic methods significantly increased completion rates for all participants (61).

For easier acceptance of PROMs, the entire care team should have a good understanding of the benefits of PROM in patient care. Also, interview administered PROMs could be used for the patients who are less likely complete the PROMs. The assessment could also be done by healthcare professionals during intake. Lastly, designing a good clinic flow will facilitate collection of PROM information.

In a preoperative setting, PROMs need to capture real- time data that will flow into a built- in algorithm to recommend actionable management plans, and include referrals that alert clinicians in a timely manner.

## Conclusion

Health care experts (62), health sector leaders (63) and CMS are consistently calling for the integration of social and functional determinants of health into patient screening for individual patient care management, value based quality performance, and as factors that should be considered to improve risk predictions for readmissions (4) and population health management (63). Ideally, PROMs in preoperative care would assess the major factors that could influence postoperative recovery, health and independence, and patients’ preferences for self-management. Integration of PROM data and well- designed electronic algorithms should be used to identify and facilitate perioperative and post-discharge care needs in real time. PROM assessments of social and functional factors as well as immediate and actionable care plans and referrals for post- discharge services are urgently needed as health systems move to surgical bundles for coronary artery bypass surgery, hip fractures, and joint replacement. Future work will focus on developing and implementing preoperative PROMs for use in health systems that are selected by CMS to implement bundle payments.

The best way to implement and evaluate the utility of PROMs in front line health care is through learning health systems. Learning health system models were first defined by the Institute of Medicine (64) and call for alignment of health system incentives with informatics and research. The best way to bring PROMS that screen for social and functional determinants of health is to align them with quality improvement programs and health system needs. In real world practice and with integrated health system’ s data warehouse, health systems could quickly evaluate the value of implementation of systematic assessments of PROMs of social and functional determinants of health. Future evaluations in learning health systems are needed to assess if screening of social and functional determinants at the point of clinical care and initiation of proactive interventions may improve surgical outcomes at the patient level (e.g., satisfaction with care, functional status, and quality of life post- surgery); the system level (e.g., length of stay, readmissions, sepsis rates); and the provider level (e.g., satisfaction with the PROMs and processes for administration to inform their clinical decision-making and efficiency).

## Figures and Tables

**Table 1:** The Association Between Depression and Various Postoperative Outcomes.

Study	Surgical Procedure	n	Outcomes
Stenman et al (15)	CABG	56,064	Mortality (HR 1.65, [1.37–1.99]); Combined end point of death or rehospitalization for myocardial infarction, heart failure, or stroke (HR 1.61, [1.38–1.89]).
Ho et al (65)	Cardiac valve surgery	648	Mortality (OR 1.90[1.07 to 3.40]).
Kerper (66)	Various	2,624	Longer LOS (5 days, IQR: 3–8 days) vs (4 days, IQR: 2–6 days) (P<0.001).
Beresnevaite (67)	CABG	109	Postoperative length of stay (beta 0.345, P<0.001) and late postoperative complications (OR 1.1 [1.02–1.19]).
Leung (12)	Major noncardiac surgery	219	Postoperative delirium incidence (P=0.048) and longer duration (P=0.027).
Caumo (16)	Abdominal elective surgery	346	Moderate to intense acute postoperative pain (OR=2.0[1.03–3.87]).
Foster (17)	Curative intent surgery for colorectal cancer	872	Self- efficacy and depression before surgery predict recovery trajectories in Quality of life, health status and wellbeing following colorectal cancer treatment.
Kazmierski (18)	Cardiac surgery with cardiopulmonary bypass	563	Preoperative major depression has OR of 4.69 [1.84– 11.93] for postoperative delirium.
Rudolph (19)	Cardiac surgery	122 derivation sample and 109 validation sample	GDS is a risk factor for postoperative delirium, RR 1.2 [1.1–1.5].
Koster (20)	Cardiac surgery	10 publications, 16,444 subjects	Depression has OR of 1.2 [1.0–1.5] to 6.3 [1.16–29.7] in predicting postoperative delirium.
Smith (21)	Noncardiac surgery	998	Preoperative executive dysfunction (OR 1.231 [1.058–1.433]) and greater levels of depressive symptoms (OR 1.370 [1.000–1.876]) were associated with postoperative delirium.
Greene (22)	Major, elective noncardiac surgery	100	Depression (OR 1.53 [1.22–2.05]) per unit of Geriatric Depression Score, and executive function, measured by the Trail Making B test (OR 1.02 per unit, [1.01–1.04]) are associated with postoperative delirium.
Smith (23)	Noncardiac surgery that require inpatient admission for a minimum of 2 days	1020	Preoperative behavioral inactivity was associated with increased risk of delirium (OR: 1.95 [1.11, 3.42]), whereas negative affect (OR: 0.65 [0.31, 1.36]) and cognitive distress (OR: 0.95 [0.63, 1.43]) were not.
